# Treatment Outcomes for Dermatosis Papulosa Nigra Using Low‐Intensity Electrodesiccation Device in African Patients

**DOI:** 10.1111/jocd.16729

**Published:** 2024-12-12

**Authors:** Frans Maruma, Ncoza Dlova, Thabiso Rafaki Petrus Mofokeng, Firas Al‐Niaimi

**Affiliations:** ^1^ School of Clinical Medicine, Faculty of Health Sciences University of the Free State Bloemfontein South Africa; ^2^ Nelson R. Mandela School of Medicine University of KwaZulu‐Natal Durban South Africa; ^3^ Consultant Dermatologist Aalborg University Aalborg Denmark; ^4^ Taktouk Clinic London UK

**Keywords:** electrocauterization, electrofulguration, seborrhoec keratoses, skin tags

## Abstract

**Objective:**

We aimed to document the treatment outcomes of African patients treated with low‐intensity electrodesiccation for DPN's. Other treatment options for DPN's include snip excision, light curettage, cryotherapy, and lasers devices such as PDL, Nd: YAG, KTP, and CO2, which are generally unsafe for darker skin types and come with high costs.

**Methods:**

Nonprobability convenience sampling of patient records was used to extract retrospective data on demographics and treatment outcomes according to the inclusion criteria. The retrospective data analysis included chi‐squared tests for associations, Kaplan–Meier survival analysis, and logistic regression analysis to examine relationships between dependent and independent variables.

**Results:**

A total of 137 patients were included in the study, with the majority being female (*n* = 129; 94.16%) compared with their male counterparts (*n* = 8; 5.84%). Most patients were under 40 years of age (*n* = 74; 54.00%), whereas the remaining were above 40 years of age (*n* = 63; 46.00%). The majority of patients had Fitzpatrick skin type V (*n* = 99; 72.26%). Adverse events such as milia (*n* = 4) and scarring (*n* = 2) were observed, but these were not associated with age, sex, or survival rate (*p* value > 0.05).

**Limitations:**

The study was conducted at a single center, which limits the ability to generalize the results.

**Conclusion:**

The low‐intensity electrodesiccation technique was found to be a safe and cost‐effective method for treating DPN's in African patients.

## 1 Introduction

First described by Dr. Aldo Castellani in 1925, dermatosis papulosa nigra (DPN) refers to benign epidermal tumors characterized by euchromatic or hyperpigmented papules, typically seen in patients with Fitzpatrick skin phototypes IV–VI [1, 2, 3]. These lesions are particularly common among individuals of African and Asian descent and tend to occur more frequently in female patients. Although asymptomatic and of no pathological concern, there is increasing demand for their removal because of aesthetic and antiaging concerns. In some cases, DPN can negatively impact a patient's quality of life [1, 4]. Some researchers classify DPN as a variant of seborrheic keratosis (SK) owing to the morphological similarities observed under both microscopic and macroscopic examination [1].

The distinction between DPN and SK is of relevant clinical importance. Although both entities are benign, there are instances where SKs may signal underlying internal malignancies. The Leser–Trélat sign, characterized by the sudden appearance of multiple eruptive SKs, is particularly noted in younger patients and is considered a paraneoplastic sign for internal malignancies, including gastrointestinal adenocarcinomas, lymphomas, and leukemia's [5, 6, 7]. This distinction between these two conditions can therefore be of clinical relevance. When DPN occurs in a patient with acanthosis nigricans, such coexistence may signal underlying endocrine disorders such as diabetes mellitus and thyroid disease. Both SK and DPN are extremely rare in the pediatric population [8, 9]. DPN lesions are typically 1–5 mm papules found on the face, neck, axillae, and upper trunk, whereas SK lesions more commonly appear on the trunk and usually present as plaques rather than papules [10, 11]. Histopathologically, both SK and DPN may exhibit features such as acanthosis, papillomatosis, and increased epidermal pigmentation [10, 11, 12, 13].

The pathogenesis of DPN remains unclear, though several theories have been proposed. One of the most compelling theories involves mutations in the fibroblast growth factor receptor‐3 (FGFR3) and phosphatidylinositol 3‐kinase (PI3K) genes [11]. This theory is supported by the observation that many patients with DPN have a family history of the condition [1, 4]. A study by Longo et al. reported a 3.1% malignancy rate in biopsies of SKs that were clinically considered to be irritated SKs [13, 14, 15]. No such data exist for DPN.

Various treatment options for DPN have been described, most with a risk when used on patients with skin of color, including a high likelihood of postinflammatory hyperpigmentation (PIH) and scarring [2, 16, 17]. There is a notable lack of data on the treatment of DPN specifically in skin of color population in South Africa. Therefore, this study retrospectively examines the outcomes of patients treated with a low‐intensity electrodesiccation device at a single aesthetic center in Bloemfontein, South Africa. The study was conducted in accordance with the principles outlined in the Declaration of Helsinki. Ethics approval was obtained from the South African Medical Association Research Ethics Committee (SAMAREC 280808016/003/2022).

## 2 Methods

This study was a retrospective, observational, single‐center investigation conducted at the Oraderm Aesthetic Clinic. The study period spanned from January 2018 to December 2021. A nonprobability convenience sampling method was used to select the medical records of all patients treated with a low‐intensity electrodesiccation device during this time frame.

### 2.1 Device Settings and Treatment Protocol

Low‐intensity was defined as settings between 1 and 3 MHz on the Conmed 2000 Hyfrecator (Utica, NY, USA) device. The device was configured to function in monopolar (mono‐terminal) mode without the use of a dispersive plate. Each dermatologic papule or nodule (DPN) was treated individually and allowed to fall off naturally within a few days posttreatment without any mechanical curettage. This technique involved using a sharp tip with slight contact to the lesion.

### 2.2 Inclusion Criteria and Follow‐Up

The study included adult patients (≥ 18 years old) whose records contained complete data and who were treated according to the following protocol.

### 2.3 Data Collection

The following key data were extracted from the medical records:

- Patient demographic details.
- Time from the procedure to complete clearance of skin or resolution of PIH.
- Procedure‐related adverse effects (e.g., scarring and PIH).
- Patient satisfaction, measured on a Likert scale from 1 to 5 (1 = not satisfied, 5 = extremely satisfied).

### 2.4 Treatment Protocol

- Preprocedure preparation: A compounded topical anaesthetic cream containing 23% lidocaine and 7% tetracaine was applied prior to the procedure.
- Postprocedure care:

- For the first 10 days posttreatment, patients applied a topical silver sulfadiazine cream twice daily.
- Starting 4 weeks after the removal of the DPNs, patients were instructed to apply a topical retinoid analog (adapalene 0.1%) at night and a 0.5% hydrocortisone cream plus SPF30+ sunscreen during the day.

### 2.5 Follow‐Up Protocol

Patients were followed‐up at intervals of 4, 12, and 24 weeks, and 1 year. The 1‐year follow‐up was conducted only for cases where PIH was not fully resolved.

### 2.6 Statistical Analysis

Descriptive statistics, including frequencies and percentages, were used to analyze the data. For continuous variables, the mean and standard deviation were calculated. The relationships between relevant variables, such as age group, skin phototype, sex, and treatment outcomes were examined using the chi‐squared test, with a significance level set at *p* < 0.05. Kaplan–Meier analysis was conducted to assess the association of age, skin phototype, and sex with the time to complete resolution of PIH. Additionally, the Log Rank (Mantel–Cox) test was used to identify factors associated with adverse effects. All statistical analyses were performed using SPSS version 22.0.

## 3 Results

### 3.1 Patients' Characteristics

A total of 137 patients were included in this analysis. The average age was 40 years (range 18–67). Most of the patients (*n* = 99; 72.26%) were of Fitzpatrick skin phototype V. See Table 1 for patient's summaries.

**TABLE 1:** Patients' characteristics (*n* = 137).

Variable name	Description	Frequency	Percentage
Sex	Female	129	94.16
	Male	8	5.84
	Total	137	100
Age group	< 40	74	54.00
	> 40	63	46.00
	Total	137	100
Skin photo type	IV	11	8.03
	V	99	72.26
	VI	27	19.71
	Total	137	100

### 3.2 Treatment Outcomes

The treatment outcomes data are summarized in Table 2.

**TABLE 2:** Patients' outcomes from the procedural and postprocedural interventions.

Variable	Description	Frequency	Percent
1‐month follow‐up: presence of PIH	Yes	137	100
	No	0	0.00
	Total	137	100
At 1‐month follow‐up, started postprocedure therapy: topical retinoid (adapalene 0.1%); 0.5% hydrocortisone plus SPF 30+	Yes	137	100
	No	0	0.00
	Total	137	100
Scarring complication 1‐year postprocedure	Yes	2	1.46
	No	135	98.54
	Total	137	100
Adverse effects of topicals (postprocedure therapy)			
Milia	Yes	4	2.92
	No	133	97.08
	Total	137	100
Atrophy	Yes	0	0
	No	137	100
	Total	137	100
Acne/Rosacea	Yes	0	0
	No	137	100
	Total	137	100
Hypopigmentation	Yes	4	2.92
	No	133	97.08
	Total	137	100
Infections	Yes	0	0
	No	137	100
	Total	137	100
Photosensitivity	Yes	0	0
	No	137	100
	Total	137	100
Documented history broad‐spectrum sunscreen (SPF30+) usage 6 months postprocedure	Yes	79	57.66
	No	58	42.34
	Total	137	100

### 3.3 Patients' Satisfaction Survey Results

**FIGURE 1:**
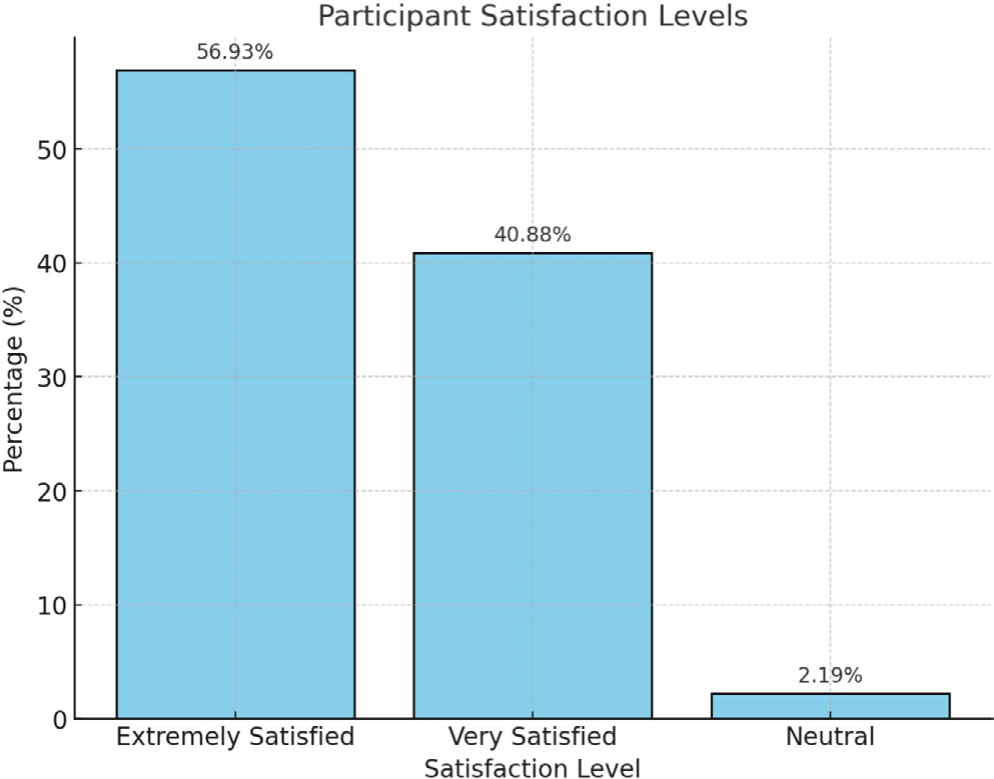
Patients' satisfaction survey results.

For patients' reported outcomes, Likert scale was used as illustrated (Figure 1).

**TABLE 3:** Descriptive statistics.

	*N*	Mean	Std. error of mean	Median	Std. deviation	Std. error of skewness	Range	Minimum	Maximum
Days from procedure to complete resolution: self‐reported to Physician‐reported	137	26.102	1.1390	23.000	13.3313	0.207	68.0	0.0	68.0
From procedure to completion resolution self‐reported	137	185.540	3.4880	180.000	40.8264	0.207	250.0	96.0	346.0
From procedure to Physician reported	137	159.438	3.3350	154.000	39.0348	0.207	275.0	61.0	336.0

### 3.4 Survival Analysis and Comparisons

See Table 4 and Figure 4.

**TABLE 4:** Comparison.

Overall comparisons			
	Chi‐squared	df	Sig.
Log Rank (Mantel–Cox)	1.359	1	0.244
Breslow (Generalized Wilcoxon)	0.418	1	0.518
Tarone–Ware	0.819	1	0.365

## 4 Discussion

DPN is a benign epidermal tumor predominantly found in individuals with skin of color, particularly those with Fitzpatrick skin types IV–VI [1, 2, 3]. Our South African‐based study population reflected this trend, with most patients being female (94.16%) with an average age of 40 years. Notably, over 70% of these patients had Fitzpatrick skin type V (Table 1). DPN typically becomes noticeable in the late 20s to early 30s. In our study, most patients presented with concerns about dark spots, which were the result of the early, small, macular lesions characteristic of DPN. The observed female predominance in our cohort aligns with findings from other studies conducted in different regions [2, 3, 5].

**FIGURE 2:**
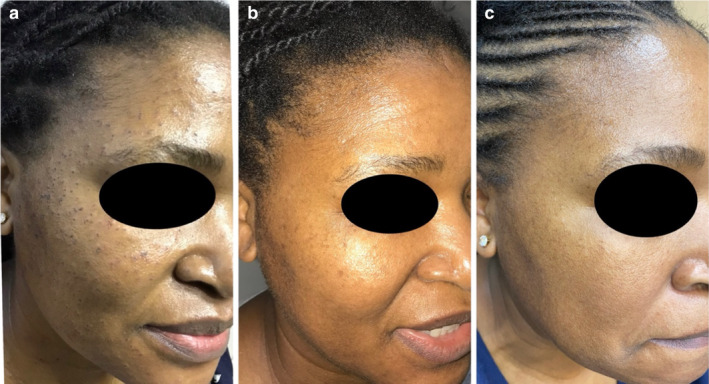
a, b, and c (day 0, month 1, and month 6).

**FIGURE 3:**
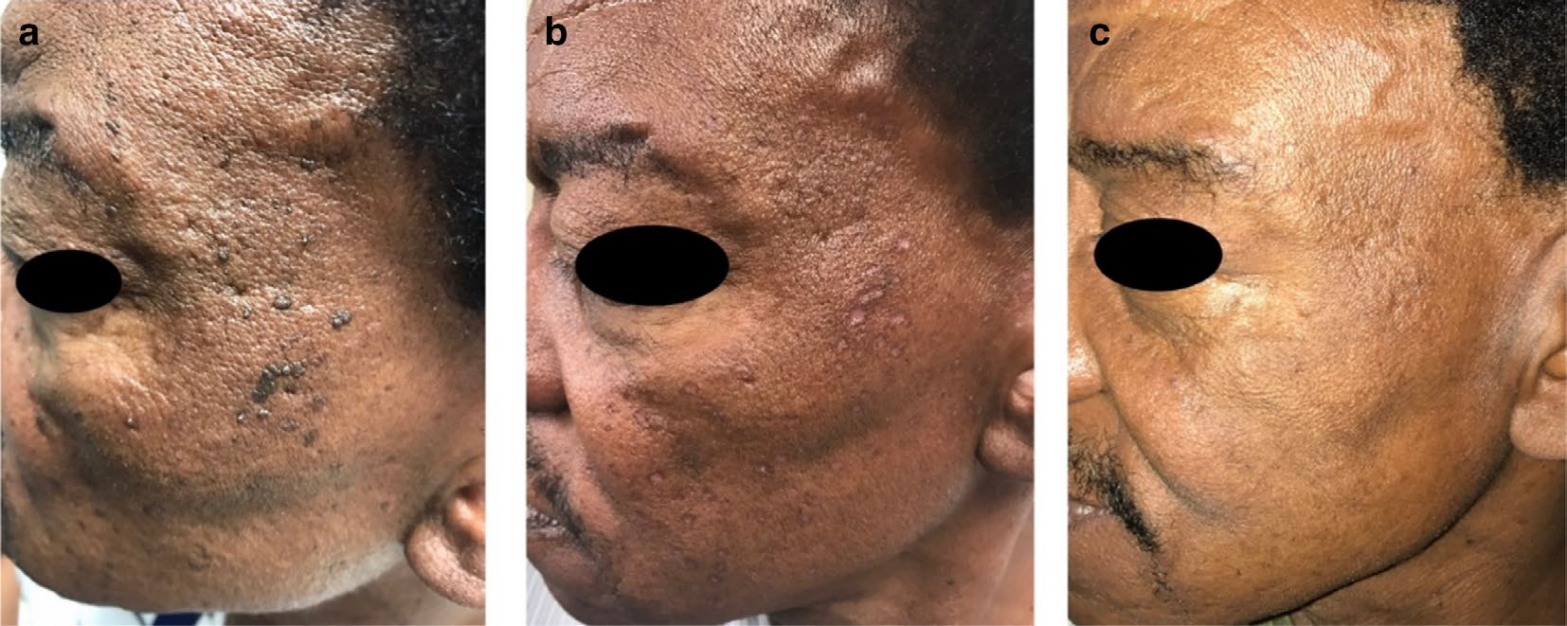
a, b, and c (day 0, month 1, and month 6).

Various treatment modalities for DPN have been documented, including snip excision, light curettage, low‐intensity electrodesiccation, cryotherapy, and laser treatments such as pulsed dye laser (PDL), long‐pulsed Nd: YAG 1064 nm, carbon dioxide ablative laser, Erbium laser, and potassium‐titanyl‐phosphate (KTP) laser [2, 16, 17, 18, 19, 20]. Although these methods have been reported to be effective, their clinical application in patients with skin of color is often limited by adverse effects such as dyspigmentation and scarring. Our study reaffirms that low‐intensity electrodesiccation can be an effective treatment for patients with skin of color, offering excellent results with minimal risk of adverse events. When asked about their satisfaction with the treatment outcomes, 56.9% of patients reported being extremely satisfied, and no reports of dissatisfaction were recorded (Figures 1, 2a‐c and 3a‐c).

### 4.1 Secondary Effects and Management: Milia and Mechanical Manipulation

A notable side effect observed in our study was the development of milia in some patients posttreatment (Table 2). Milia are small, white cysts that can form because of skin trauma or occlusion. Our analysis indicated no significant association between the occurrence of milia and patient factors such as age, sex, or skin type. To minimize the formation of milia, we recommend an aftercare regimen that includes noncomedogenic moisturizers and sunscreen in the morning, as well as a nightly topical retinoid to prevent occlusion and support skin cell turnover. On the basis of our findings, we suspect that the use of topical corticosteroids might have contributed to milia formation in our cohort, highlighting the need for careful selection of aftercare products. In our cohort, there were two cases of scarring 1‐year postprocedure. The scarring occurred on nonfacial areas, notably the neck and upper chest (Table 2). As part of good clinical practice, the patients should be advised to avoid mechanical manipulation of the treated areas. Clinicians should also be cautious when treating scar‐prone areas such as the upper chest and pre‐counsel the patients appropriately to manage expectations.

### 4.2 Managing Postinflammatory Hyperpigmentation in DPN Treatment

One of the primary concerns when treating DPN in skin of color is the risk of postinflammatory hyperpigmentation (PIH), a condition in which dark patches develop at the site of skin injury or inflammation. PIH can be distressing for patients, particularly when it affects visible areas such as the face. In our study, all participants (*n* = 137) experienced PIH one‐month posttreatment, underscoring the inevitability of this side effect in darker skin types. However, with appropriate pretreatment counseling, patients understood that PIH would likely be transient, helping them manage expectations.

The mean time for PIH to resolve completely was 159 days according to physician assessments and 185 days on the basis of patient self‐reports (Table 3). This slight discrepancy between physician and patient reports may stem from subjective perceptions of pigmentation changes or differences in lighting and assessment environments. PIH resolution is typically gradual, with complete fading within 6 months to a year. Educating patients on the natural course of PIH and its expected duration can help prevent dissatisfaction and ensure adherence to aftercare instructions.

In terms of managing PIH, we found that a posttreatment skincare regimen including daily use of broad‐spectrum sunscreen and moisturizing creams helped improve skin recovery. Postinflammatory hyperpigmentation is a common, though often transient, complication observed after various dermatological procedures as observed in 100% (*n* = 137) of our cohort (Table 2). The literature highlights several strategies that may help mitigate or manage PIH, with treatment outcomes varying based on individual factors and the modalities used. Among these approaches are chemical peels, retinoid analogs, and triple combination therapy (Kligman's formula), which incorporates hydroquinone, a retinoid, and a corticosteroid for synergistic effects. Additionally, growth factors derived from platelet‐rich plasma (PRP) have shown promise in promoting skin regeneration and potentially minimizing pigmentation.

Nonhydroquinone alternatives, such as arbutin, cysteamine, niacinamide, and tranexamic acid, offer additional options for addressing hyperpigmentation, particularly in cases where hydroquinone is unsuitable or contraindicated [21, 22]. These agents may help to suppress melanin production through various mechanisms, such as inhibiting tyrosinase activity or reducing melanocyte activation.

To optimize outcomes, these interventions can be employed in combination with a broad‐spectrum sunscreen that has a minimum of SPF30. Sunscreen plays a critical role in protecting the skin from UV radiation, which exacerbates PIH, and in stabilizing melanocytes [22]. The authors recommend preprocedure regimens that incorporate such agents to prepare the skin by reducing baseline inflammation, stabilizing melanocyte activity, and enhancing the skin's resilience during treatments like electrodesiccation. Postprocedure, these measures not only protect against external triggers of pigmentation but also promote faster clearance of PIH by modulating melanin synthesis and encouraging skin repair. It is the opinion of authors that combining preventive and corrective strategies, that could help reduce both the severity and duration of pigmentation while improving overall patient outcomes.

### 4.3 Association Between Demographic Data and Various Outcomes

We applied the chi‐squared test to assess the association between demographic variables—such as age group, skin phototype, and sex—and various outcomes. The results indicated a significant association between the use of SPF 30+ sunscreen and an average age of < 40 years. The majority of patients (60; 44%) reported consistent use of SPF 30 sunscreen during the 6 months postprocedure (*p* < 0.0001). Notably, a substantial proportion of patients under 40 years old (53; 39%) expressed extreme satisfaction with the treatment outcomes (*p* < 0.0001). However, no significant associations were found between skin phototype and the various outcomes (*p* > 0.05), nor between sex and the other outcomes (*p* > 0.05).

**FIGURE 4:**
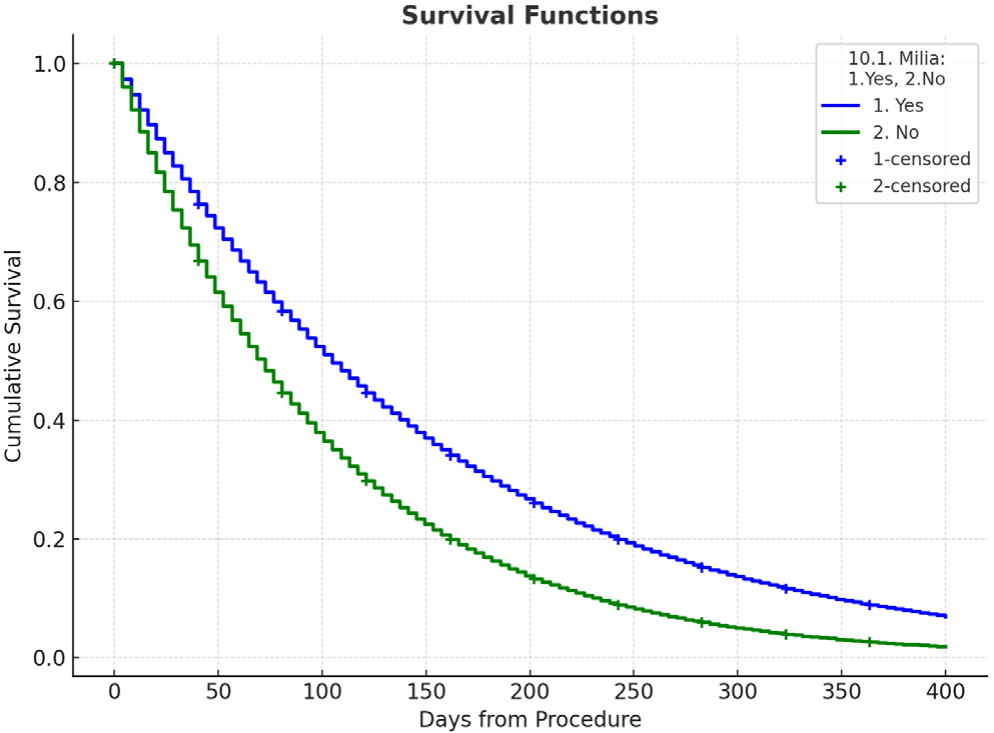
Kaplan–Meier analysis for patients with and without milia.

We also conducted a Kaplan–Meier analysis to assess if there was any association between age, skin type, and sex with the time from the procedure to complete resolution (Figure 4). The log‐rank (Mantel‐Cox) analysis showed that the occurrence of milia as a side effect was not significantly associated with skin phototype, age, or sex, as indicated by a *p* value > 0.05. Additionally, the time to resolution, which showed no significant association with age, sex, or skin phototype, was found to be less than a year, with a survival rate of 20% (Table 4).

### 4.4 Limitations and Future Directions

Although our study provides valuable insights into DPN treatment in a South African cohort, there are limitations to consider. First, the study was conducted in a single‐center setting with a predominantly female cohort, which may limit the generalizability of findings to other populations or to male patients. Additionally, although our treatment protocol minimized complications such as scarring and prolonged PIH, it may not be applicable to all skin types or lesion severities. Future studies could explore the efficacy of alternative treatments, including fractional lasers or topical agents, to expand the therapeutic arsenal for DPN specifically in South African patients. Moreover, investigating the genetic and molecular basis of DPN could offer insights into targeted therapies and preventive measures.

## 5 Conclusion

In conclusion, DPN is a common, benign skin condition in individuals with darker skin tones that often prompts treatment for cosmetic reasons. To our knowledge, this study is the first and largest to report on the outcomes of low‐intensity electrodesiccation in South African patients with skin of color. The majority of participants were female, with skin phototype V being the most common, and an average age of 40 years. Our study affirms the effectiveness of low‐intensity electrodesiccation for DPN, particularly in patients with Fitzpatrick skin types IV–VI, where the risk of PIH and scarring is a primary concern. Appropriate pretreatment counseling, careful execution of the procedure, and appropriate aftercare measures were key to achieving high patient satisfaction with minimal complications. Although PIH was a universal side effect in our study, its transient nature and eventual resolution underscore the importance of managing patient expectations.

Through careful selection of treatment modalities and attention to aftercare, clinicians can offer safe and effective solutions for DPN that cater specifically to the needs of patients with skin of color. Further research is warranted to continue refining treatment protocols, minimize side effects, and explore the underlying causes of DPN to better serve affected populations.

## Author Contributions

F.M. planned, designed, conceptualized, managed data, drafted, proofread, and approved the final manuscript. N.D. conceptualized, designed, managed data, proofread, and approved final manuscript. T.R.P.M. assisted with conceptualization, data management, drafting of results discussion, and final manuscript approval. F.A.‐N. conceptualized, reviewed data analysis, interpretation, discussion, proofread, and approved the final manuscript.

## Ethics Statement

This study was conducted in accordance with the principles of the Declaration of Helsinki. Ethical approval was obtained from the South African Medical Association Ethics Committee (SAMAREC 280808016/003/2022).

## Consent

Written informed consent was obtained from respective patients for the purpose of this publication.

## Conflicts of Interest

The authors declare no conflicts of interest.

## Data Availability

The data that support the findings of this study are available on request from the corresponding author. The data are not publicly available due to privacy or ethical restrictions.
